# Pattern of Water Use and Seed Yield under Terminal Drought in Chickpea Genotypes

**DOI:** 10.3389/fpls.2017.01375

**Published:** 2017-08-09

**Authors:** Jiayin Pang, Neil C. Turner, Yan-Lei Du, Timothy D. Colmer, Kadambot H. M. Siddique

**Affiliations:** ^1^School of Agriculture and Environment, The University of Western Australia Perth, WA, Australia; ^2^The UWA Institute of Agriculture, The University of Western Australia Perth, WA, Australia; ^3^State Key Laboratory of Grassland Agroecosystems, Institute of Arid Agroecology, School of Life Sciences, Lanzhou University Lanzhou, China

**Keywords:** flower abortion, fraction of transpirable soil water, plant water use pattern, pod abortion, reproductive growth and development, transpiration

## Abstract

Drought, particularly terminal drought, reduces the yield of chickpea (*Cicer arietinum* L.). Terminal drought tolerance and water use patterns were evaluated under controlled conditions in 10 genotypes of desi chickpea. Withholding water from early podding reduced vegetative growth, reproductive growth, seed yield, and water use efficiency for seed yield in all genotypes. The genotype Neelam, which produced the highest seed yield when water was withheld, used the least water when well-watered; however, its aboveground biomass at maturity did not differ significantly from six of the nine other genotypes. Indeed, the water-stressed Neelam had the lowest daily transpiration rate during the early stages of water stress and the highest during the later stages, thereby maintaining the highest soil water content in the first 16 days after water was withheld, which enabled higher pod production, lower pod abortion, and better seed filling. Genotypes differed in the threshold value of the fraction of transpirable soil water when flowering and seed set ceased in the water-stress treatment. We conclude that a conservative water use strategy benefits seed yield of chickpea exposed to water shortage during early podding.

## Introduction

Water shortage during the reproductive phase, termed “terminal drought,” is a major constraint to the yield of chickpea (*Cicer arietinum* L.) whether crops are grown on stored soil moisture in subtropical areas or on current rainfall in Mediterranean climatic regions (Siddique and Sedgley, 1986, 1987; Leport et al., 1999; Kashiwagi et al., 2015). Global chickpea production is estimated to be reduced by 33% by drought stress (Kashiwagi et al., 2015). Drought can dramatically reduce final seed yield as a result of reduced flower and pod production, increased flower and pod abortion, and reduced seed size (Leport et al., 1998, 1999; Davies et al., 1999; Fang et al., 2010). A field study of 108 chickpea genotypes showed a two-fold range in yield when grown in a water-limited Mediterranean-type climate, indicating variation in drought tolerance (Pang et al., 2017) which was further evaluated for selected genotypes in the present controlled-environment experiment.

The pattern of water extraction contributes to yield determination in crops grown with limited soil water, particularly in indeterminate crops such as, chickpea, as the maintenance of transpiration and C-fixation during the seed-filling period is crucial for yield (Merah, 2001; Kato et al., 2008; Zaman-Allah et al., 2011b). Stomata progressively close as the soil dries to slow further water loss (Sadras and Milroy, 1996; Leport et al., 1999; Kholova et al., 2010; Fang et al., 2011). In pearl millet (*Pennisetum glaucum* L.), under well-watered conditions, drought-tolerant genotypes had lower transpiration rates per unit leaf area than drought-sensitive genotypes; and this “constitutive water use trait” correlated positively with yield during terminal drought as water conserved when plentiful was available for grain filling as water became scarce during terminal drought (Kholova et al., 2010). Similarly, when water was withheld from 23 days after sowing (DAS) when plants were at the vegetative stage, drought-tolerant chickpea genotypes used less water than drought-sensitive genotypes during the vegetative stage attributed to lower canopy conductance thereby conserving water in the soil profile for use during seed filling (Zaman-Allah et al., 2011b). It is unknown whether the two-fold yield variation among the 108 chickpea genotypes in our previous field studies (Pang et al., 2017) was associated with different water use patterns.

When water is withheld, transpiration, photosynthesis, leaf water relations, leaf expansion, flowering, or seed set remain unaffected as the soil dries until a threshold soil water content is reached; at which point, that can differ depending on the process, each begin to decrease (Ray and Sinclair, 1998; Liu and Stützel, 2002; Zaman-Allah et al., 2011b; Kong et al., 2015; Pushpavalli et al., 2015; Pang et al., 2017). Genotypic differences in the proportion of plant available water in the soil [the fraction of transpirable soil water (FTSW)] when transpiration first begins to decrease (i.e., threshold FTSW) have been found in chickpea (Pushpavalli et al., 2015), pearl millet (Kholova et al., 2010), soybean (*Glycine max* L.; Hufstetler et al., 2007), and peanut (*Arachis hypogaea* L.; Bhatnagar-Mathur et al., 2007). In chickpea, threshold FTSW-values for the decrease in transpiration were lower in most drought-sensitive genotypes compared with drought-tolerant genotypes under outdoor conditions (Zaman-Allah et al., 2011a), but not under glasshouse conditions (Zaman-Allah et al., 2011a; Pushpavalli et al., 2015). These studies identified the threshold FTSW-values for a reduction in transpiration, but not for other processes. In a glasshouse experiment with two chickpea genotypes, Pang et al. (2017) showed that seed set ceased at FTSW threshold values of 0.57 in both genotypes, although the plants continued to produce flowers and pods until the FTSW reached ~0.16. As far as we are aware, Pang et al. (2017) is the only study to investigate the threshold values of FTSW for reproductive processes in chickpea, but data are needed on a wider set of genotypes to determine whether the FTSW threshold values for reproductive processes differ among genotypes and whether the FTSW threshold is a useful parameter to discriminate between drought-tolerant and drought-sensitive genotypes.

The present glasshouse experiment grew 10 chickpea genotypes, with contrasting seed yields at three dryland field sites in south-western Australia, in large (106 kg) containers of soil to investigate the effects of terminal drought imposed at early podding on reproductive processes. The objectives were to determine the effects of terminal drought on: (1) reproduction, including both flower and pod production and abortion, seed set, seed size, and seed yield; (2) the patterns of water use; and (3) threshold values of FTSW for the cessation of flower and seed set.

## Materials and methods

### Plant material and growth conditions

Ten desi chickpea genotypes, two commercial cultivars (Neelam and Genesis836), and eight breeding lines with similar phenology, were selected on the basis of yield data from 108 genotypes in 2012 and 62 genotypes in 2013 grown at three dryland field sites (Pang et al., 2017). Three of the selected genotypes (DICC8172, WACPE2160, CICA1229) had consistently high yield rankings, four (DICC8156, DICC8218, DICC9073, CICA0912) had consistently low yield rankings, and three (Genesis836, Neelam, DICC9100) had variable yield rankings across the three sites (Table 1). The pedigrees of the 10 genotypes are given in Table 1. The experiment was carried out from May to November 2014 in a temperature-controlled glasshouse in Perth, Australia (31.57°S, 115.47°E) with 23 and 13°C average maximum and minimum air temperatures and a mean relative humidity of 59%.

**Table 1:** Pedigree, ranking for yield at three field sites in 2012 and 2013, and phenology of 10 chickpea genotypes used in the glasshouse experiment.

**Genotype**	**Pedigree**	**Yield ranking out of 108 genotypes at York in 2012**	**Yield ranking out of 62 genotypes at Bindi in 2013**	**Yield ranking out of 62 genotypes at Cunderdin in 2013**	**Time to 50% flowering (DAS)**	**Time to 50% podding (DAS)**	**Days from 50% flowering to 50% podding**	**Time to physiological maturity in well-watered plants (DAS)**	**Time to physiological maturity in water-stressed plants (DAS)**
DICC8172	Genesis836/PBG5	6	13	2	70 ± 3	96 ± 1	26	166 ± 1	144 ± 1
WACPE2160	8627P-2/ICC13729	14	3	5	81 ± 1	97 ± 0	16	167 ± 1	145 ± 1
CICA1229	99226^*^02HS001/CICA0604	2	5	3	69 ± 3	94 ± 1	25	166 ± 1	147 ± 2
Genesis836	BDN 9-3^*^K1184/ICP87440	na	16	44	72 ± 3	95 ± 1	23	167 ± 1	141 ± 0
Neelam	8511-19/ICC13729	85	37	4	76 ± 2	95 ± 1	19	159 ± 6	143 ± 1
DICC9100	ICC12004/Moti	4	45	6	77 ± 2	97 ± 0	20	164 ± 1	142 ± 1
DICC8156	ICCV96836/PBG 5	67	57	61	81 ± 1	96 ± 1	15	164 ± 1	147 ± 0
DICC8218	ICCV96836/ICC12004	102	43	57	78 ± 1	98 ± 0	20	166 ± 1	148 ± 3
DICC9073	ICCV/ICCV04516	105	42	46	79 ± 1	97 ± 0	18	165 ± 0	143 ± 1
CICA0912	98081-3024/CICA0512	na	61	55	78 ± 0	97 ± 0	19	160 ± 4	144 ± 1

*Phenological data are means ± s.e.m. of eight replicates for plants grown in the glasshouse in 2014. DAS = days after sowing. na indicates “not available” as these genotypes were not included at York. The actual yields of the field results are presented in Pang et al. (2017)*.

Plants were grown in 80 L containers with dimensions of 460 × 470 mm at the top, 290 × 190 mm at the bottom and a height of 770 mm (Sulo, Somersby, NSW, Australia). Each container had five 12-mm diameter drainage holes and held (bottom upwards): 5 kg of coarse gravel, nylon mesh, 105.6 kg of (well-mixed) four parts of sieved, dried soil, and one part river sand (soil depth was 630 mm). The soil was a reddish-brown sandy clay loam (clay = 27%, silt = 9%, sand = 64%), classified as Red Calcic Dermosal (Isbell, 1996), from the upper 0.15 m at the site of the 2013 field experiment at Cunderdin (31.64°S, 117.24°E) in Pang et al. (2017). The field soil contained 6 μg g^−1^ nitrate-N, 3 μg g^−1^ ammonium-N, 46 μg g^−1^ Colwell P, 691 μg g^−1^ Colwell K, and had a pH (CaCl_2_) of 7.1. Diammonium phosphate (18% N and 20% P) at 0.016 μg g^−1^ soil was mixed into the soil:sand mixture before filling the containers. The soil in the containers had a bulk density of 1.60 g cm^−3^.

Two days before sowing, all containers were watered to 80% of field capacity (FC), which was 16 L per container. The water content at FC was 19% (w/w; Pang et al., 2017). The containers were weighed using a custom-made balance (Pang et al., 2017).

Eight containers were used for each genotype. On 22 May 2014, 15 seeds (coated with P Pickel-T®; 360 g L^−1^ thiram, 200 g L^−1^ thiabendazole) were planted in each container at 25 mm depth with about 10 g of peat-based Group N rhizobium (New Edge Microbials, Albury, NSW, Australia). Seedlings were thinned to five plants at 18 DAS to give a similar density to that in field, and the soil surface was covered with a 30-mm layer of plastic beads to minimize soil evaporation. All containers were watered to 80% FC by weighing every 2 days until the two watering treatments were imposed. The experiment had two water treatments and four replicates.

### Water treatments

Two watering treatments were imposed at 100 DAS when the plants were at early podding; for each genotype: (1) four containers were kept well-watered (WW) to 80% FC by watering to weight every 2 days until 144 DAS when most of the water-stressed plants had reached maturity; and (2) four containers had watering withheld to maturity (water stress, WS). Volumetric soil water content at different depths was monitored using a portable soil water monitoring probe (Diviner2000, Sentek Sensor Technologies, Stepney, SA, Australia), inserted via a 1.0-m high, 50 mm diameter vertical access tube installed in the middle of each container (Pang et al., 2013). The probe was calibrated against soil samples for which water content was measured by drying at 105°C in 1.0-m pots (15 mm in diameter) filled with the same soil mixture as that in the experiment (data not shown; *R*^2^ = 0.99).

### Estimation of the fraction of transpirable soil water (FTSW)

FTSW-values represent the fraction of remaining soil water available for transpiration on each day of the experiment. The difference in container weight when plants were watered to 80% FC prior to the start of the WS treatment and that when transpiration had become negligible in the WS containers provides the total transpirable soil water (TTSW; Pushpavalli et al., 2015). Daily FTSW = (daily container weight – final container weight)/(initial container weight – final container weight; Ray and Sinclair, 1998). FTSW for every 2 days of the experiment was back-calculated at the end of the experiment. FTSW-values are presented between 1 (80% FC at the whole container level) and 0 (at the end of drought stress 42 days after water was withheld when water loss was negligible). In addition, for the soil water probe measurements, FTSW at each depth = (daily volumetric soil water content – final volumetric soil water content)/(initial volumetric soil water content – final volumetric soil water content) at each soil depth. Both the initial and final volumetric soil water contents differed at the different soil depths.

### Flower and pod tagging

The start of flowering and podding was recorded for plants in each container, and the time to 50% flowering and 50% podding was recorded when ≥3 plants per container had at least one open flower or one pod (3 mm long and visible outside the corolla). All new flowers and pods on one randomly-selected plant in each container were tagged every 2 days with the date of flowering and podding noted on the tags.

### Harvest procedure

At physiological maturity when the pods had turned brown and dry, all plants were cut at soil level and partitioned into leaves + stems (bulked together) and pods. The plant used for flower and pod tagging was harvested separately while the other four plants per container were combined. For the four untagged plants per container, the seeds were threshed from the pods and dried at 30°C for 7 days and weighed, while the leaves + stems + pod wall (shell) were bulked together and dried at 60°C for 48 h and weighed. For the tagged plant, all pods with the same podding date were combined then for each date separated into pod wall and seeds for counting, and weighed after oven-drying at 30°C for 7 days. The information on the tags enabled total numbers of flowers, pods, flower abortion, and abscised pods to be determined. All pods were checked for seeds and empty pods had no seed or contained a seed <40% of the average size (Leport et al., 2006). Harvest index (HI) was calculated as the ratio of seed dry weight to aboveground dry weight. The container means of aboveground dry weight, seed yield and HI were calculated based on the data from the four untagged plants plus the tagged plant (i.e., data from all five plants per container).

### Statistical analysis

The experiment was a two-factorial (genotype and water treatment) randomized complete block design with four replicates. Four blocks with 20 containers for each block (with 10 genotypes and two water treatments) were arranged in rows in the glasshouse. Data for growth and other parameters were analyzed by general analysis of variance (ANOVA) in Genstat version 15.2 (Lawes Agricultural Trust, Rothamsted Experimental Station, UK, 2007). The statistical model with FTSW as the explanatory variable used a split-line regression in Genstat to assess FTSW threshold values for cumulative flower number and cumulative seed number. Linear regressions between seed yield and water use in Figure **5** were fitted and analyzed in SigmaPlot Version 13.0.

## Results

### Phenology

Although chosen for their similar phenologies in the field, the time to first flower varied from 62 DAS in CICA1229 to 75 DAS in DICC8156 (*P* < 0.001), while their corresponding time to 50% flowering ranged from 69 DAS to 81 DAS (*P* < 0.001; Table 1). Podding in the 10 genotypes commenced from 89–94 DAS (*P* = 0.296) and time to 50% podding ranged from 94 DAS in CICA1229 to 98 DAS in DICC8218 (*P* < 0.01; Table 1). The first flowers in all genotypes and both water treatments failed to produce a pod. The interval from 50% flowering to 50% podding ranged from 15–16 days in the late-flowering genotypes to 25–26 days in the early-flowering genotypes. Within genotypes, flowering and podding times did not differ between plants later assigned to the WW and WS treatments (data not shown). Among the 10 genotypes, the WS plants reached physiological maturity at 141–148 DAS which was 16–26 days earlier than the WW plants (159–167 DAS). Physiological maturity in the WW plants was enforced by cessation of watering at 144 DAS.

### Change of soil water content

The volumetric soil water content increased with soil depth (*P* < 0.001; Table 2). At the start of the water treatments at 100 DAS, the volumetric soil water content at 80% FC increased from 0.19 m^3^ m^−3^ in the 0–0.1 m soil layer to 0.31 m^3^ m^−3^ at 0.5–0.6 m soil depth. The volumetric soil water content remained similar at all depths in the WW treatment. By contrast in the WS treatment, the soil water content decreased in all soil layers, but when further daily water loss was negligible (that is FTSW = 0, as defined) at 42 days after water was withheld (DAWW), the volumetric soil water content was 0.04 m^3^ m^−3^ in 0–0.1 m soil layer and still 0.10 m^3^ m^−3^ at 0.5–0.6 m (Table 2). The change from the beginning to the end of the WS treatment ranged from 0.15 m^3^ m^−3^ in the upper soil to 0.20 m^3^ m^−3^ in the bottom 0.1 m soil depth (Table 2). The 10 genotypes did not differ in the initial or final volumetric soil water contents at different depths (data not shown, *P* > 0.05).

**Table 2:** Volumetric soil water content at the beginning (FTSW = 1) and end (FTSW = 0) of the water-stress (WS) treatment at different soil depths.

**Soil depth (m)**	**Beginning of WS treatment when FTSW = 1.0 (m^3^ m^−3^ soil)**	**End of WS treatment when FTSW = 0 (m^3^ m^−3^ soil)**	**Difference between the beginning and end of WS treatment (m^3^ m^−3^ soil)**
0–0.1	0.19 ± 0.00	0.04 ± 0.00	0.15 ± 0.00
0.1–0.2	0.23 ± 0.00	0.07 ± 0.00	0.15 ± 0.00
0.2–0.3	0.25 ± 0.00	0.08 ± 0.00	0.16 ± 0.00
0.3–0.4	0.25 ± 0.00	0.09 ± 0.00	0.16 ± 0.00
0.4–0.5	0.27 ± 0.00	0.09 ± 0.00	0.17 ± 0.00
0.5–0.6	0.31 ± 0.00	0.10 ± 0.00	0.20 ± 0.00

*Data are means ± s.e.m. of 10 genotypes with four containers per genotype (n = 40). Significant effects of soil depth on the volumetric soil water content at the start of WS treatment (P < 0.001, LSD_0.05_ = 0.006 m^3^ m^−3^), the end of WS treatment (P < 0.001, LSD_0.05_ = 0.003 m^3^ m^−3^), and the difference between the start and the end of the WS treatment (P < 0.001, LSD_0.05_ = 0.005 m^3^ m^−3^); no effects of genotype or the interaction between genotype × soil depth were found (P > 0.05)*.

**Figure 1:**
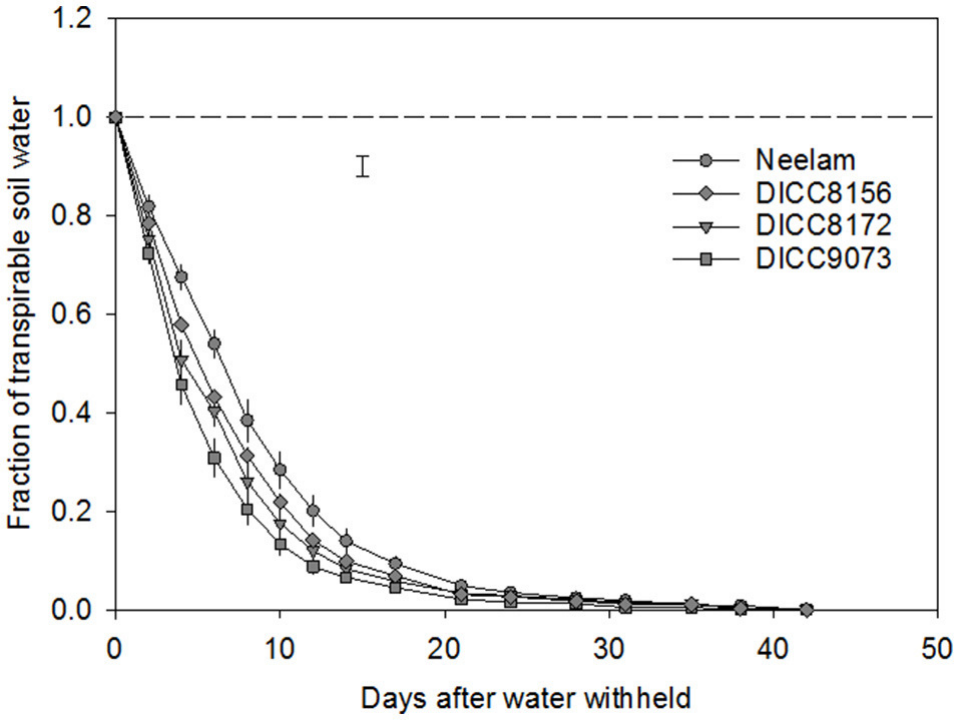
Change in the fraction of transpirable soil water (FTSW) in the water-stressed (WS) treatment with time after the start of water treatments (100 DAS) in four chickpea genotypes: Neelam, DICC8156, DICC8172, and DICC9073. The dashed line represents FTSW in the well-watered (WW) treatment, which was maintained at 1.0 by watering to 80% field capacity every 2 days. The bar represents the least significant difference (LSD) at *P* = 0.05 for the interaction between genotype × days of treatment in the WS treatment. Data are means ± s.e.m. (*n* = 4). The data for the other six genotypes are in the Figure S1.

**Figure 2:**
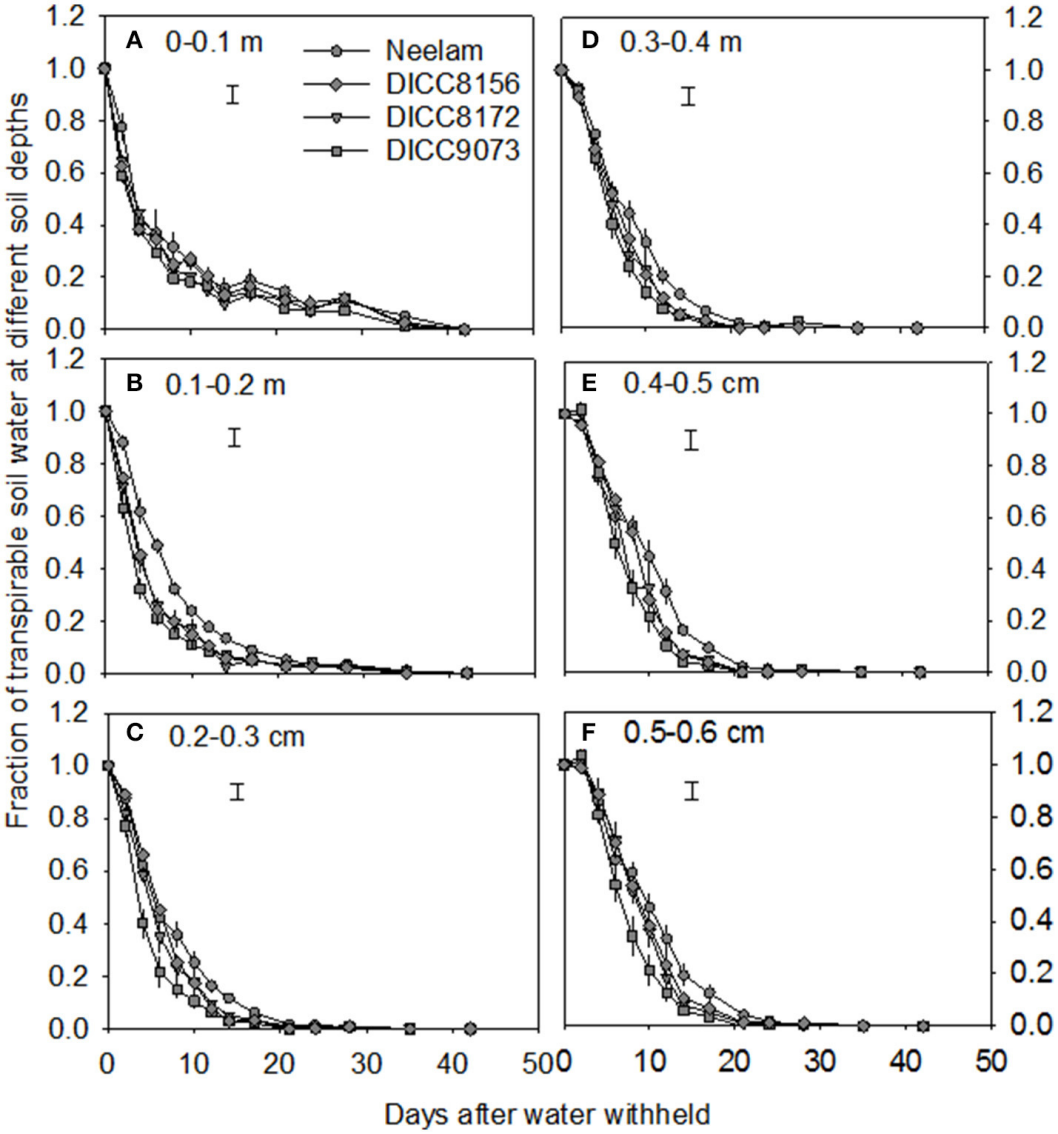
Volumetric soil water content at six different soil depths 0–0.1 m **(A)**, 0.1–0.2 m **(B)**, 0.2–0.3 m **(C)**, 0.3–0.4 m **(D)**, 0.4–0.5 m **(E)**, and 0.5–0.6 m **(F)** with time after the start of the water treatments (100 DAS) in the water-stressed treatment in four chickpea genotypes: Neelam, DICC8156, DICC8172, and DICC9073. Bars at each soil depth represent the least significant difference (LSD) at *P* = 0.05 for the interaction between genotype × days of treatment in the WS treatment. Data are means ± s.e.m. (*n* = 4). The data for the other six genotypes are in the Figure S2.

**Figure 3:**
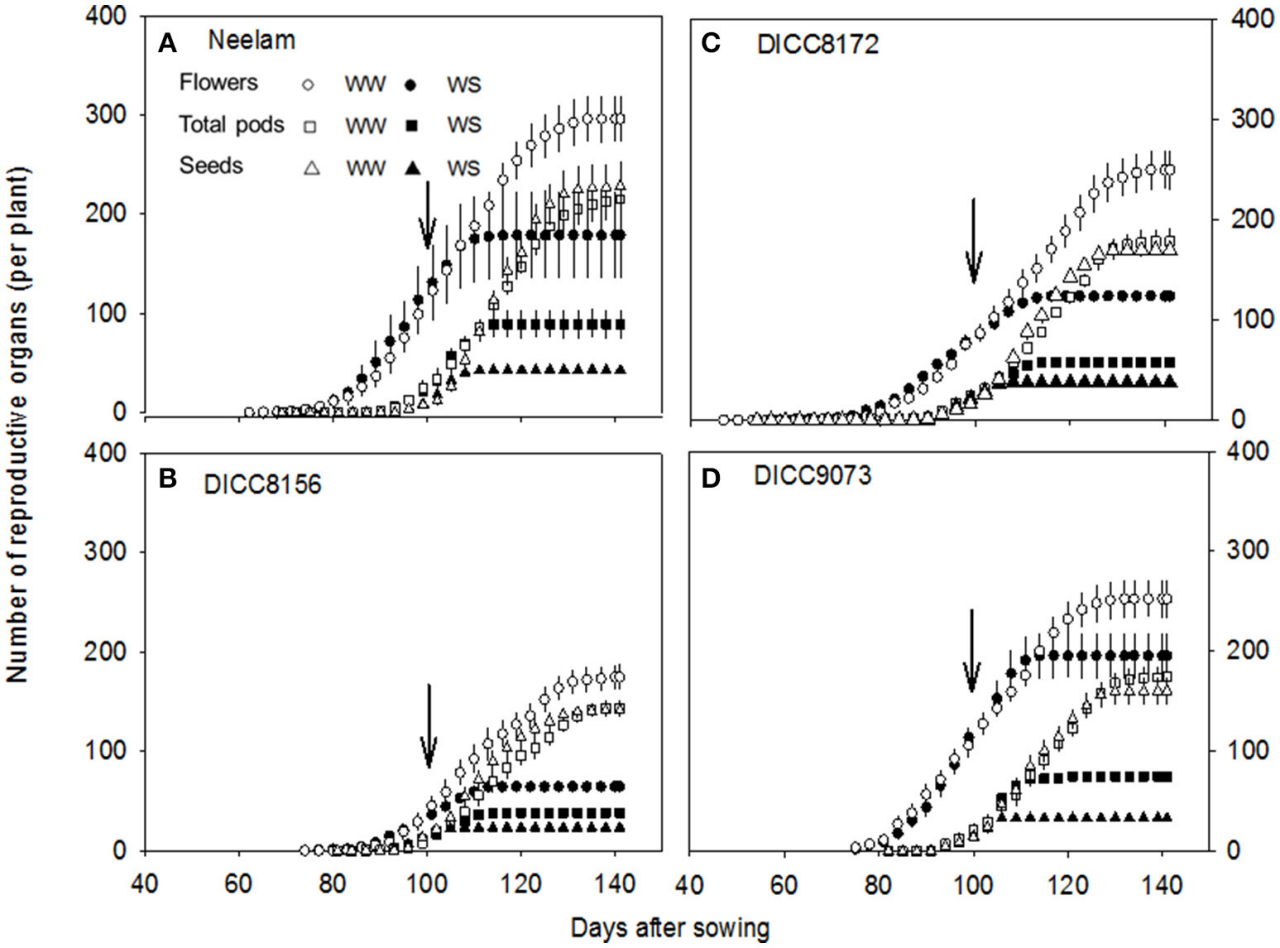
Changes in the cumulative number of flowers, total pods, and seeds per plant with time (days) after sowing in the well-watered (WW) and water-stressed (WS) treatments in four chickpea genotypes: Neelam **(A)**, DICC8156 **(B)** DICC8172 **(C)**, and DICC9073 **(D)**. Data are means ± s.e.m. (*n* = 4). Arrows indicate the start of the water treatments. The data for the other six genotypes are in the Figure S3.

In the WS treatment, the rates of water use varied among genotypes, that is there was a significant interaction between genotype × days of treatment for FTSW (*P* < 0.001; Table 3, Figure 1 and Figure S1). For clarity, four genotypes including those with the slowest (Neelam) and fastest (DICC9073) water use after water was withheld (DAWW), and two genotypes (DICC8156 and DICC8172) from our previous study (Pang et al., 2017) are shown in Figure 1 (and similarly in Figures 2, 3), while the other six genotypes with the intermediate rates of water use are shown as supplementary data (Figure S1, and similarly in Figures S2, S3). The imposition of WS reduced FTSW steadily in all 10 genotypes for the first 14 DAWW (Figure 1 and Figure S1). At 14 DAWW, FTSW ranged from 0.07 in DICC9073 to 0.14 in Neelam (*P* < 0.05), after which it slowly decreased with no significant differences among genotypes. From 31 DAWW water use was minimal in all 10 genotypes (Figure 1 and Figure S1). FTSW in the WW treatment was maintained near 1.0 by watering the containers every 2 days to 80% FC. In the WS treatment, FTSW at each soil depth followed a similar trend to that at the container level (Figure 2 and Figure S2), indicating that roots were present and active in water uptake at all soil depths.

**Table 3:** Significance of different sources of variability for a range of parameters.

	**G**	**D**	G^*^D
Fraction of transpirable soil water (FTSW) after water was withheld	^***^	^***^	^***^
Change in FTSW with days after water withheld at 0–0.1 m	^**^	^*^	^**^
Change in FTSW with days after water withheld at 0.1–0.2 m	^***^	^***^	^**^
Change in FTSW with days after water withheld at 0.2–0.3 m	^***^	^***^	^***^
Change in FTSW with days after water withheld at 0.3–0.4 m	^***^	^***^	^*^
Change in FTSW with days after water withheld at 0.4–0.5 m	^***^	^***^	^***^
Change in FTSW with days after water withheld at 0.5–0.6 m	^***^	^***^	^**^
**Parameters at maturity**	**G**	**Trt**	G*Trt
Aboveground dry weight (g plant^−1^)	^***^	^***^	^*^
Seed yield (g plant^−1^)	^**^	^***^	^**^
Harvest index	^***^	^***^	ns
Number of total flowers (plant^−1^)	^***^	^***^	ns
Number of aborted flowers (plant^−1^)	^***^	^***^	^**^
Percentage of flower abortion	^***^	^***^	^*^
Number of total pods (plant^−1^)	^***^	^***^	^**^
Number of abscised pods (plant^−1^)	^***^	ns	ns
Percentage of abscised pods	^***^	^***^	^***^
Number of filled pods (plant^−1^)	^***^	^***^	^*^
Number of empty pods (plant^−1^)	^***^	^***^	^*^
Percentage of empty pods	^***^	^*^	ns
Number of seeds (plant^−1^)	^***^	^***^	^**^
Seed number per filled pod	^**^	^***^	ns
Mean seed weight (g seed^−1^)	^***^	^***^	^**^
Total water use before water treatment (L plant^−1^)	^***^	ns	ns
Total water use (L plant^−1^)	^***^	^***^	^**^
Water use efficiency (g grain L^−1^ H_2_O)	^***^	^***^	^*^
Threshold value of fraction of transpirable soil water when flower development ceased	^***^	–	–
Threshold value of fraction of transpirable soil water when seed development ceased	^***^	–	–

Significant effects are indicated for genotype (G), water treatment (Trt), days of treatment (D), and their interactions (ns, no significant difference;

* P < 0.05;

** P < 0.01;

*** *P < 0.001). Trt were well-watered (WW) and water-stressed (WS) imposed from early podding*.

### Aboveground dry weight, yield, HI and seed size

Total aboveground dry weight ranged from 75.7 to 101.9 g plant^−1^ in the WW treatment, whereas in the WS treatment the range was from 34.6 to 41.3 g plant^−1^, a reduction of 54–62% as a result of the WS treatment (Table 4). Seed yield in the WW treatment ranged from 25.6 to 33.8 g plant^−1^, while in the WS treatment the range was 2.0 to 5.8 g plant^−1^ (Table 4), yields in the WS treatment varied from 6% of the WW controls in CICA0912 to 21% of the controls in Neelam. Ranking the genotypes from the highest to the lowest seed yield in the WS treatment (Table 4), or on yields in the WS treatment relative to those in the WW treatment (the rankings were similar), shows that Neelam and DICC8172 were the most drought-tolerant genotypes, while CICA0912 was the most drought-sensitive genotype. HI varied with genotype (*P* < 0.001) and decreased markedly in the WS treatment (*P* < 0.001), but the two-way interaction was not significant (Tables 3, 4). In the WW treatment the HI ranged from 0.28 in CICA0912 to 0.38 in Neelam and WACPE2160, and decreased in the WS treatment to 0.05 in CICA0912 and 0.17 in Neelam (*P* < 0.001; Table 4).

**Table 4:** Aboveground dry weight, seed yield, harvest index, yield components, percentage flower abortion, percentage abscised pods, and percentage empty pods, total water use and water use efficiency for grain (WUE_G_) for 10 chickpea genotypes in the well-watered (WW) and water-stressed (WS) treatments at physiological maturity.

		**Aboveground dry weight (g plant^−1^)**	**Seed yield (g plant^−1^)**	**Harvest index**	**Percentage flower abortion**	**Number of total pods (plant^−1^)**	**Number of filled pods (plant^−1^)**	**Percentage of abscised pods**
Neelam	WW	81.3 ± 7.4	27.27 ± 2.69	0.38 ± 0.02	27 ± 4	214 ± 18	154 ± 20	6 ± 4
	WS	35.0 ± 3.2	5.76 ± 0.29	0.17 ± 0.02	50 ± 6	89 ± 4	36 ± 2	21 ± 4
DICC8172	WW	84.5 ± 3.1	29.58 ± 0.58	0.35 ± 0.01	28 ± 2	172 ± 15	123 ± 5	11 ± 1
	WS	36.1 ± 0.3	5.17 ± 0.27	0.12 ± 0.02	54 ± 2	58 ± 2	30 ± 1	26 ± 5
WACPE2160	WW	75.7 ± 3.2	28.83 ± 0.93	0.38 ± 0.01	25 ± 3	141 ± 8	109 ± 3	8 ± 3
	WS	34.7 ± 1.5	5.07 ± 0.85	0.15 ± 0.02	52 ± 1	59 ± 8	30 ± 3	30 ± 2
Genesis836	WW	84.8 ± 2.3	29.75 ± 2.35	0.35 ± 0.02	30 ± 1	221 ± 14	135 ± 7	10 ± 2
	WS	41.3 ± 1.0	4.57 ± 0.25	0.11 ± 0.01	61 ± 1	97 ± 1	32 ± 4	39 ± 5
DICC9073	WW	84.7 ± 4.2	29.99 ± 2.22	0.35 ± 0.01	31 ± 1	175 ± 11	117 ± 10	17 ± 4
	WS	37.8 ± 1.2	4.13 ± 0.43	0.09 ± 0.01	62 ± 1	75 ± 3	27 ± 1	33 ± 3
DICC8156	WW	92.1 ± 2.5	33.79 ± 2.23	0.37 ± 0.02	19 ± 3	153 ± 11	115 ± 8	12 ± 2
	WS	40.3 ± 0.9	3.91 ± 0.21	0.08 ± 0.01	41 ± 3	38 ± 2	19 ± 2	24 ± 4
CICA1229	WW	77.1 ± 3.8	25.63 ± 1.77	0.33 ± 0.01	27 ± 3	193 ± 16	118 ± 4	8 ± 2
	WS	34.6 ± 2.6	3.79 ± 0.42	0.11 ± 0.02	52 ± 3	75 ± 15	25 ± 4	32 ± 2
DICC8218	WW	96.7 ± 7.6	29.99 ± 2.07	0.31 ± 0.02	33 ± 1	237 ± 27	143 ± 8	16 ± 2
	WS	38.0 ± 1.2	3.43 ± 0.24	0.07 ± 0.02	69 ± 3	72 ± 4	23 ± 2	38 ± 3
DICC9100	WW	88.4 ± 2.1	27.69 ± 1.10	0.31 ± 0.01	31 ± 4	197 ± 12	125 ± 6	8 ± 2
	WS	39.1 ± 2.0	3.22 ± 0.33	0.07 ± 0.01	65 ± 2	50 ± 6	22 ± 2	30 ± 3
CICA0912	WW	101.9 ± 7.9	32.62 ± 3.91	0.28 ± 0.02	29 ± 1	211 ± 13	151 ± 21	4 ± 1
	WS	38.9 ± 2.1	2.01 ± 0.31	0.05 ± 0.01	63 ± 5	51 ± 6	16 ± 1	40 ± 5
LSD_0.05_	Genotype	6.8	2.50	0.03	6	24	14	5
	Water	3.0	1.12	0.01	3	11	6	2
	Genotype × Water	9.6	3.54	ns	8	34	20	8
Neelam	WW	22 ± 4	229 ± 25	1.5 ± 0.0	145 ± 6	10.4 ± 0.3	28.8 ± 1.5	1.06 ± 0.09
	WS	39 ± 6	43 ± 2	1.2 ± 0.0	134 ± 1	10.3 ± 0.9	13.1 ± 0.9	0.44 ± 0.03
DICC8172	WW	18 ± 4	168 ± 7	1.4 ± 0.0	177 ± 8	12.9 ± 0.2	33.8 ± 1.1	0.88 ± 0.03
	WS	22 ± 4	36 ± 3	1.2 ± 0.0	146 ± 5	12.1 ± 0.3	14.8 ± 0.3	0.35 ± 0.02
WACPE2160	WW	15 ± 4	157 ± 4	1.4 ± 0.0	182 ± 6	11.3 ± 0.7	30.1 ± 0.9	0.96 ± 0.02
	WS	20 ± 5	41 ± 4	1.4 ± 0.1	123 ± 3	11.3 ± 0.4	14.1 ± 0.4	0.36 ± 0.06
Genesis836	WW	29 ± 7	204 ± 16	1.5 ± 0.0	146 ± 5	11.9 ± 0.3	31.2 ± 0.7	0.90 ± 0.06
	WS	29 ± 2	42 ± 3	1.3 ± 0.2	110 ± 11	12.2 ± 0.4	15.3 ± 0.4	0.30 ± 0.01
DICC9073	WW	16 ± 4	160 ± 14	1.4 ± 0.1	186 ± 8	12.8 ± 0.7	33.4 ± 1.0	0.90 ± 0.06
	WS	31 ± 3	33 ± 1	1.2 ± 0.0	125 ± 7	12.7 ± 0.2	15.4 ± 0.2	0.27 ± 0.03
DICC8156	WW	15 ± 3	141 ± 11	1.2 ± 0.0	222 ± 6	13.0 ± 0.9	31.9 ± 1.0	1.07 ± 0.10
	WS	26 ± 1	21 ± 3	1.1 ± 0.0	173 ± 11	12.6 ± 0.4	14.9 ± 0.3	0.25 ± 0.02
CICA1229	WW	31 ± 3	169 ± 9	1.4 ± 0.0	152 ± 10	11.9 ± 0.9	31.4 ± 1.2	0.81 ± 0.03
	WS	35 ± 5	32 ± 7	1.3 ± 0.1	122 ± 8	11.7 ± 0.7	14.3 ± 0.7	0.27 ± 0.03
DICC8218	WW	24 ± 4	187 ± 11	1.3 ± 0.1	151 ± 2	11.2 ± 0.4	33.1 ± 1.7	0.91 ± 0.04
	WS	21 ± 2	26 ± 3	1.1 ± 0.0	137 ± 16	11.2 ± 0.4	13.9 ± 0.4	0.25 ± 0.02
DICC9100	WW	28 ± 2	178 ± 12	1.4 ± 0.0	156 ± 5	11.7 ± 0.7	32.1 ± 0.6	0.86 ± 0.04
	WS	26 ± 5	26 ± 5	1.2 ± 0.1	128 ± 3	11.3 ± 0.6	14.0 ± 0.6	0.23 ± 0.02
CICA0912	WW	25 ± 3	210 ± 29	1.4 ± 0.0	156 ± 9	12.2 ± 0.7	37.4 ± 1.9	0.87 ± 0.07
	WS	32 ± 4	17 ± 0	1.0 ± 0.0	139 ± 3	12.0 ± 0.8	14.2 ± 0.8	0.14 ± 0.02
LSD_0.05_	Genotype	8	20	0.2	14	1.2	1.7	0.08
	Water	4	9	0.1	6	ns	0.8	0.04
	Genotype × Water	ns	29	ns	19	ns	2.4	0.11

*Data are means ± s.e.m. (n = 4). For the parameters where genotype × water treatment interaction is significant, LSD-values at P = 0.05 are given for the interaction; if a two-way interaction is not significant, the LSD_0.05_-values are given for the effects of genotype and/or water. ns, no significant difference. Data for aboveground dry weight, seed yield, harvest index, water use, and WUE_G_ from five plants per container were pooled and the mean per container presented, while data for the remaining parameters were the means of the tagged plants*.

Water stress reduced seed size (mean seed weight; *P* < 0.001, Tables 3, 4). Mean seed weight in the WS treatment was 8–11% less for Neelam, DICC8218, and CICA0912, but these did not significantly differ from those of the WW treatment (*P* > 0.05), whereas in the remaining seven genotypes, mean seed weight decreased by 18–33% (*P* < 0.001, Table 3) compared with the WW controls (Table 4).

### Flower production and abortion

**Figure 4:**
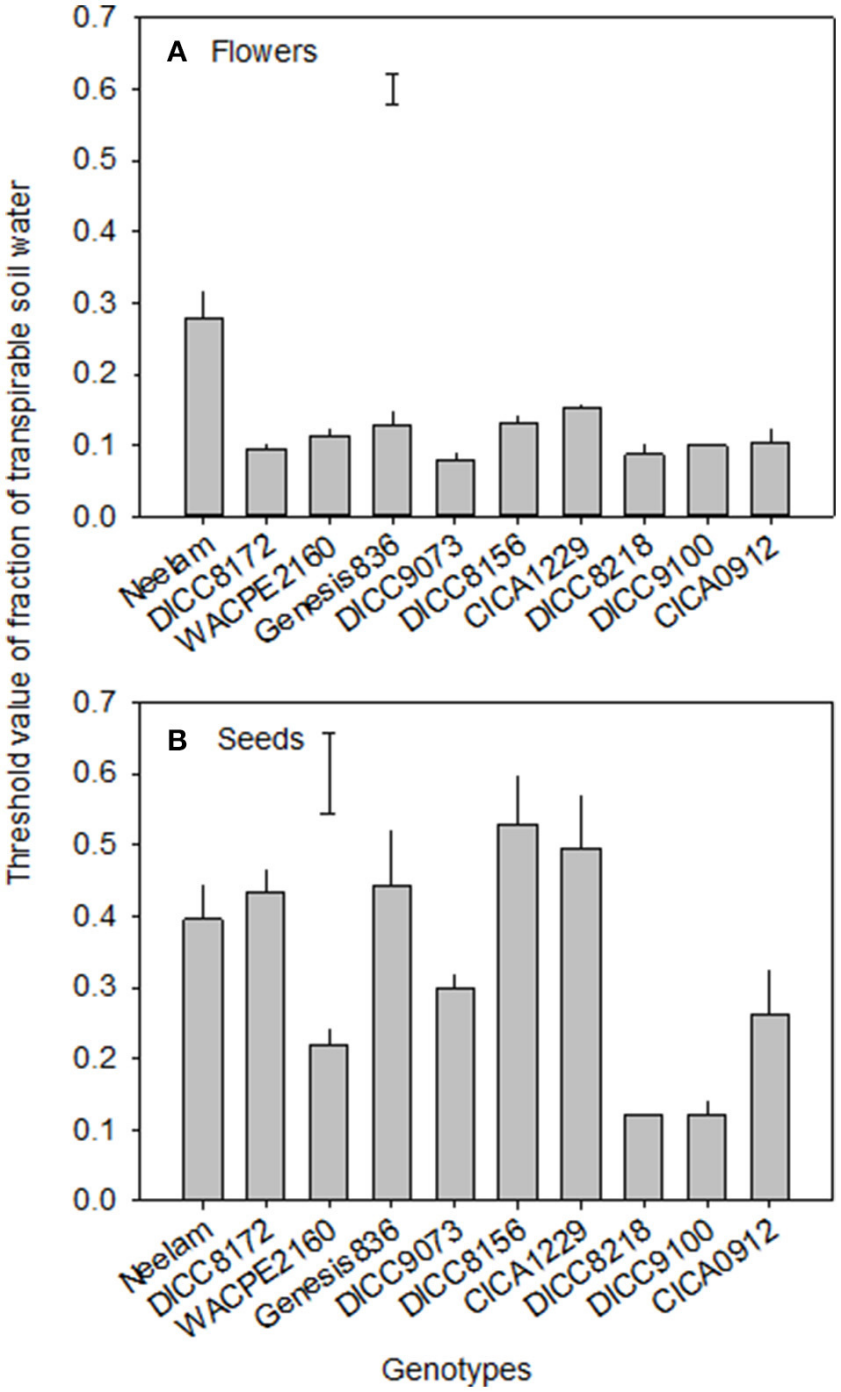
The values of the fraction of transpirable soil water (FTSW) at which flowering **(A)** and seed set **(B)** ceased in 10 chickpea genotypes in the WS treatment (terminal drought). Data are means ± s.e.m. (*n* = 4). Bars represent least significant difference (LSD) at *P* = 0.05 among genotypes.

At the start of the water treatments (100 DAS), the number of flowers already produced per plant varied among the 10 genotypes (*P* < 0.001), from 37 in DICC8156 to 154 in Genesis836 (Figure 3 and Figure S3). The total number of flowers produced per plant varied with genotype (*P* < 0.001, Table 3) and water treatment (*P* < 0.001, Table 3, Figure 3 and Figure S3). In total, WW plants produced 174–354 flowers plant^−1^; the WS treatment reduced these numbers by 20% in Genesis836 to 67% in DICC8156 (*P* < 0.001). In the WS treatment, although flowering stopped earlier than the WW controls in all genotypes, flower production continued to increase at the same rate (the number of flowers produced per day) as in the WW controls for several days in many of the genotypes (e.g., Neelam), but slowed shortly after water was withheld in a few genotypes (e.g., DICC8156 and DICC 8172; Figure 3 and Figure S3). As the soil dried in the WS treatment, flower number continued to increase linearly in Neelum until 10 DAWW (110 DAS) when flower production ceased at the FTSW of 0.28, while flower production continued in the other genotypes until FTSW decreased to 0.08–0.15 at 11–14 DAWW (111–114 DAS; Figure 4A and Figure S4). No correlation was found between the FTSW when flowering ceased and the time to 50% flowering (*R*^2^ = 0.02, *P* > 0.05). Flowering in the WW controls ceased 34–40 days after the two water treatments were imposed (134–140 DAS), despite soil water content being maintained at 80% FC (Figure 3 and Figure S3). Averaging the WW and WS treatments, DICC8218 and Genesis836 had the most flowers, followed by Neelam and DICC9073, while DICC8156 had the least, but there was no significant interaction between genotype and water treatment (*P* > 0.05, Table 3, Figure 3 and Figure S3).

There was a significant interaction between genotype and water treatment for the number of aborted flowers (*P* < 0.01) and percentage flower abortion (*P* < 0.05, Tables 3, 4 and Table S1). The number of aborted flowers in the WS treatment increased compared with that in the WW treatment for Genesis836, DICC9073, and DICC8218, but not in the other seven genotypes (Table S1). However, in the WS treatment, the 10 genotypes always had a higher percentage flower abortion than in the WW treatment, ranging from 80 to 165% higher (Table 4).

In the WW treatment, seed yield was positively correlated with flower number (*R*^2^ = 0.33, *P* < 0.001), but not with percentage flower abortion (Table 5). In the WS treatment, there was no correlation between seed yield and flower number, while there was a negative linear correlation between seed yield and percentage flower abortion (*R*^2^ = 0.24, *P* < 0.01; Table 5). No correlation among the genotypes was found between seed yield and FTSW when flowering ceased (*R*^2^ = 0.14, *P* > 0.05).

**Table 5:** The relationship between seed yield (SY) and aboveground dry weight (ADW), number of flowers (FN), number of filled pods (FP), number of seeds (SN), seed size (SS, mean seed weight), percentage flower abortion (PFA), and percentage of abscised pods (PAP) in the well-watered and water-stressed treatments.

	**Linear relationship for well-watered**	**Linear relationship for water-stressed**
Aboveground dry weight (ADW)	SY = −0.475 + 0.338 ADW *R*^2^ = 0.82^***^	SY = −0.650 + 0.123 ADW *R*^2^ = 0.43^***^
Number of flowers (FN)	SY = 14.243 + 0.0541 FN *R*^2^ = 0.33^***^	*R*^2^ = 0.09 ns
Number of filled pods (FP)	SY = 3.704 + 0.195 FP *R*^2^ = 0.78^***^	SY = −0.675 + 0.180 FP *R*^2^ = 0.90^***^
Number of seeds (SN)	SY = 6.122 + 0.130 SN *R*^2^ = 0.80^***^	SY = −0.515 + 0.1443 SN *R*^2^ = 0.89^***^
Seed size (SS)	*R*^2^ = 0.01 ns	SY = −3.434 + 63.471 SS *R*^2^ = 0.43^***^
Percentage flower abortion (PFA)	*R*^2^ = 0.06 ns	SY = 11.35 – 0.121 PFA *R*^2^ = 0.24^**^
Percentage of abscised pods (PAP)	*R*^2^ = 0.02 ns	SY = 9.145 – 0.138 PAP *R*^2^ = 0.42^***^

*The equations are the fitted linear regression with the correlation coefficients. The red font represents negative correlations*.

** P < 0.01;

*** *P < 0.001; ns, no significant difference*.

### Pod and seed production, abscised pods and empty pods

At the start of the two water treatments (100 DAS), pods plant^−1^ differed among the 10 genotypes (*P* < 0.001), ranging from 7 ± 2 in DICC8218 to 32 ± 3 in Genesis836 (Figure 3 and Figure S3). Pod production ceased 12–15 DAWW (112–115 DAS) for all 10 genotypes; the rate of pod production in WS plants during these first 12–15 days ranged from 1.9 pods day^−1^ in DICC8156 to 5.0 pods day^−1^ in Genesis836 and DICC9073. At maturity, the total number of pods plant^−1^ in the WS treatment ranged from 38 in DICC8156 to 97 in Genesis836, that is, from ~25% of those in the WW plants for DICC8156 (153) and DICC9100 (197), to ~43% of that in the WW plants in Genesis836, Neelam, DICC9073 and WACPE2160 (221, 214, 175, and 141, respectively; Table 4, Figure 3 and Figure S3); this genotype × water treatment interaction was significant (*P* < 0.01, Table 3).

Of the pods that were set before the water treatments were imposed, the number that developed into filled pods (0–16 plant^−1^) and seeds (0–20 plant^−1^) differed among genotypes in both the WW and WS treatments (Figure 3 and Figure S3). Further, there was a significant genotype × water treatment interaction for the number of filled pods (*P* < 0.05) and seeds (*P* < 0.01) at the final harvest. The number of filled pods plant^−1^ in the WS treatment varied from 16 in CICA0912 to 36 in Neelam, 11–28% of the 109 to 154 filled pods plant^−1^ in the WW controls (Table 4). Similarly, the WS treatment decreased the number of seeds plant^−1^ to 17 in CICA0912 to 43 in Neelam, a reduction of 79–92% of the 141–229 seeds plant^−1^ in the WW controls (Tables 3, 4). Seed production rates in the WS plants ranged from 1.0 seed day^−1^ in CICA0912 to 4.0 seeds day^−1^ in Neelam and Genesis836 (Figure 3 and Figure S3), but seed set ceased 5–12 DAWW (105–112 DAS) when the FTSW was ~0.5 in DICC8156 and CICA1229 (at 5 DAWW) and 0.12 in DICC8218 and DICC9100 (at ~12 DAWW; *P* < 0.001, Table 3, Figure 4B and Figure S4). In the WW treatment 39% of the pods had two seeds per pod compared with only 20% in the WS treatment, which contributed to the 14% reduction in average seed number per filled pod over the 10 genotypes (*P* < 0.001), but there was no significant genotype × water treatment interaction (*P* > 0.05, Tables 3, 4).

Genotypes differed significantly in the number of abscised pods (*P* < 0.001, Table 3 and Table S1), but water treatment and the genotype × water treatment interaction were not significant *(P* > 0.05, Table 3). The WS treatment increased (*P* < 0.001) the percentage of abscised pods from the 4 to 17% of the number of abscised pods in the WW controls to twice these percentages in DICC8172, DICC8156, and DICC9073, and to 10 fold higher in CICA0912 (Table 4).

The number of empty pods plant^−1^ decreased in the WS treatment compared to the WW treatment (*P* < 0.05, Table 3 and Table S1), due to the reduced total pod number in the WS treatment. While the absolute numbers of empty pods plant^−1^ decreased, WS increased the percentage of empty pods to total pods in most of the genotypes; the exceptions were DICC9100, Genesis836, and DICC8218 (*P* < 0.05; Table 4). Averaging the WW and WS treatments, Neelam and CICA1229 had the highest percentage of empty pods, followed by Genesis836 and CICA0912, while WACPE2160 had the lowest percentage (*P* < 0.001). There was no significant genotype × water treatment interaction for the percentage of empty pods (*P* > 0.05, Table 3).

Seed yield was positively associated with the number of seeds plant^−1^ and with number of filled pods in both the WW (*R*^2^ = 0.80, *P* < 0.001; *R*^2^ = 0.78, *P* < 0.001) and WS (*R*^2^ = 0.89, *P* < 0.001; *R*^2^ = 0.90, *P* < 0.001) treatments (Table 5). However, seed yield was only positively correlated with seed size in the WS treatment (*R*^2^ = 0.43, *P* < 0.001; Table 5). Seed yield was negatively associated with the percentage of abscised pods in the WS treatment (*R*^2^ = 0.42, *P* < 0.001), but not in the WW treatment (Table 5). No correlation was observed between seed yield and the FTSW when seed set ceased (*R*^2^ = 0.06, *P* > 0.05).

### Total water use and water use efficiency in the production of seed yield

Before the imposition of the water treatments, a significant difference was found in the amount of total water used among the genotypes (*P* < 0.001); Neelam had used the least water (10.4 L plant^−1^), followed by DICC8218, while DICC8172, DICC9073, and DICC8156 used the most (~12.6 L plant^−1^), but water use did not differ significantly between plants that were assigned to the WW or WS treatment within each genotype (Tables 3, 4). After the imposition of water treatments, water use continued to differ among genotypes in the WW treatment, ranging from 18.4 L plant^−1^ in Neelam to 25.2 L plant^−1^ in CICA0912, but was similar (2.6–2.8 L plant^−1^, *P* > 0.05) among genotypes in the WS treatment (Table 3). Thus, the total water use from sowing to maturity in the WS plants of the 10 genotypes was 13.1 L plant^−1^ in Neelam to 15.4 L plant^−1^ in DICC9073, while the corresponding values in the WW plants varied greatly from 28.8 L plant^−1^ in Neelam to 37.4 L plant^−1^ in CICA0912 (Table 4).

Whole-life-span water use efficiency for seed production (WUE_G_) in the WW treatment varied from 1.06 g L^−1^ in Neelam and DICC8156 to 0.81 g L^−1^ in CICA1229; while the corresponding values in the WS treatment ranged from 0.44 g L^−1^ in Neelam to 0.14 g L^−1^ in CICA0912. There was a significant genotype × water treatment interaction for WUE_G_ (*P* < 0.01; Table 3), ranging from a 59% reduction in Neelam to an 84% reduction in CICA0912 compared with the WW treatment (Table 4).

**Figure 5:**
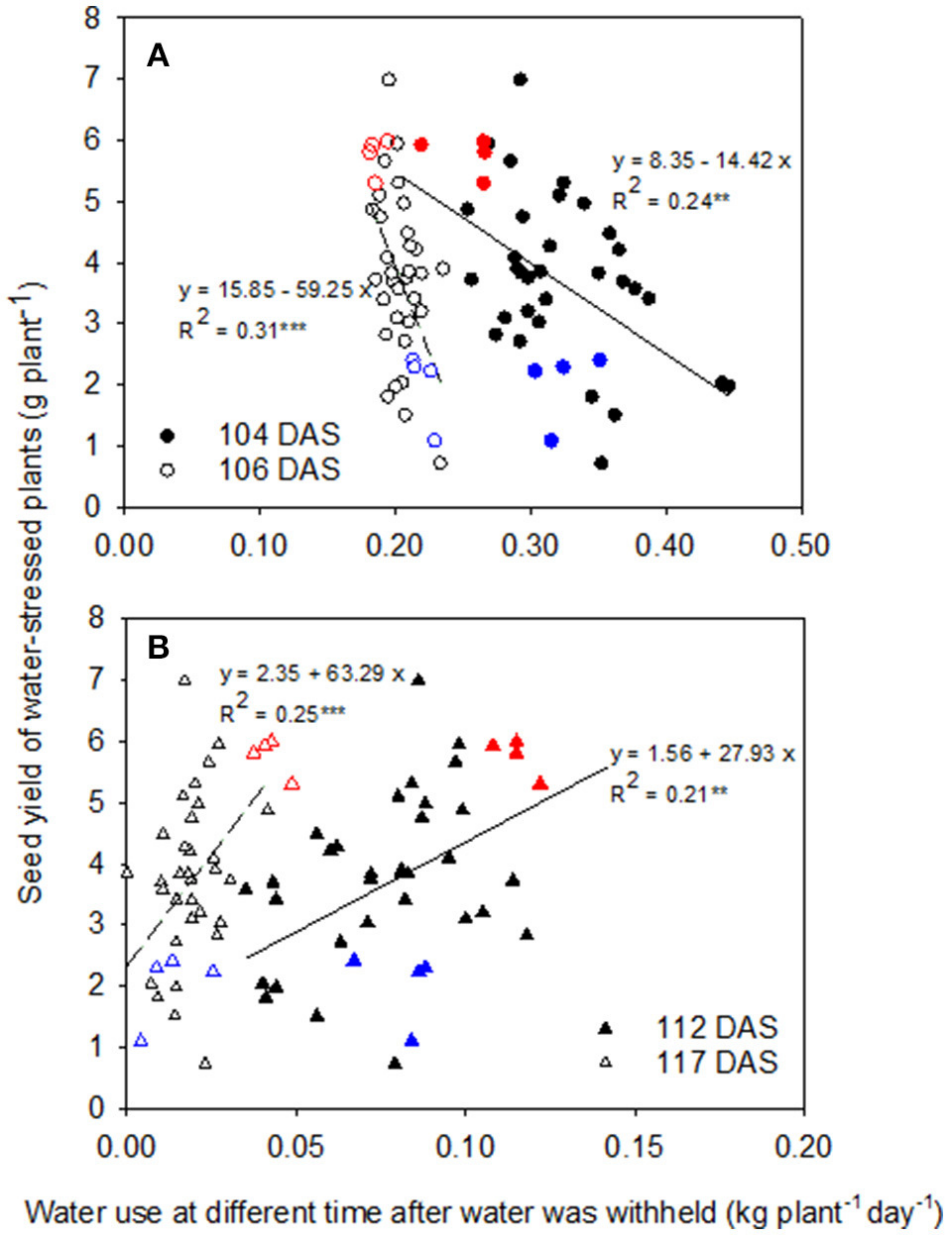
Relationships between seed yield and water use in the water-stressed (WS) treatment at 4 and 6 days after water was withheld (DAWW) (104 and 106 days after sowing (DAS), respectively, **A**), and 12 and 17 DAWW (112 and 117 DAS, respectively, **B**) in 10 chickpea genotypes. The fitted linear regressions with regression coefficients (*R*^2^) are given. ^**^P < 0.01; ^***^P < 0.001. Red and blue symbols represent Neelam and CICA0912, respectively.

Among genotypes, seed yield showed weak negative correlations with daily water use after water was withheld for 4 days (104 DAS, *R*^2^ = 0.24, *P* < 0.01) and 6 days (106 DAS, *R*^2^ = 0.31, *P* < 0.001; Figure 5A), while there were weak positive correlations with daily water use when water had been withheld for 12 days (112 DAS, *R*^2^ = 0.21, *P* < 0.01) and 17 days (117 DAS, *R*^2^ = 0.25, *P* < 0.001; Figure 5B), implying that water conservation at the start of the water-stress treatment enabled greater water use that benefitted yield later as the water shortage became more severe. At the early stage of the WS treatment (4 and 6 DAWW), Neelam (highest seed yield under WS) transpired less water per day than CICA0912 (lowest seed yield under WS), while the opposite difference was observed during the later stages of WS (12 and 17 DAWW).

## Discussion

Terminal drought imposed from early podding reduced shoot growth, reproductive growth, seed yield, and WUE_G_ in all 10 desi chickpea genotypes selected for their differences in yield under water-limited dryland field conditions. Among the 10 genotypes, Neelam had the highest seed yield and WUE_G_ when exposed to terminal drought in this glasshouse study. When adequately watered, Neelam used the least water among the 10 genotypes both prior to the imposition of the WS treatment and over the entire growth period. However, the aboveground biomass of Neelam at maturity did not differ significantly from that of six of the nine other genotypes in the WW treatment and from all others in the WW treatment. After water was withheld, all of the genotypes under WS used similar total amounts of the soil water, but the pattern of water use differed, with Neelam having the lowest daily transpiration rate during the early stages of the WS treatment and the highest during the later stages. Thus, Neelam maintained the highest FTSW among the 10 genotypes until 16 DAWW. Our results suggest that the higher yield of Neelam under terminal drought was due to a more favorable pattern of water use, i.e., a more conservative water use when water is plentiful and during the early stage of WS, thereby leaving more soil water available for pod production and seed filling during the later period. The study also revealed genotypic variation in FTSW threshold values at which flower and seed set ceased, but these parameters were not correlated with seed yield. The implications of these and other findings are discussed.

### Genotypic difference in the pattern of water use under water stress (Ws)

This study demonstrated a negative linear correlation (*R*^2^ = 0.24 at 4 DAWW and 0.31 at 6 DAWW) between seed yield and the daily rate of transpiration during the early stages of WS (0–6 days after water was withdrawn), but a positive linear correlation (*R*^2^ = 0.21 at 12 DAWW and 0.25 at 17 DAWW) between these parameters during the later stages (10–21 DAWW). While these correlations are not very strong, they are significant so we conclude that when water is withheld during the reproductive stage, slower water use during the early reproductive phase resulted in more water available during the later reproductive phase when podding and seed filling are occurring, thereby contributing to the higher yields observed in Neelam when exposed to terminal drought. These results support and extend an earlier finding by Zaman-Allah et al. (2011b) where water was withheld from 23 DAS for 20 chickpea genotypes; seed yield was negatively correlated with water use between 28 and 38 DAS (*R*^2^ ≈ 0.25), but positively correlated with water use between 48 and 61 DAS (*R*^2^ ≈ 0.4). Our current findings make an important addition to the understanding of chickpea drought tolerance. While the earlier study by Zaman-Allah et al. (2011b) found that seed yield was positively correlated with the amount of water taken up during reproductive growth when chickpea was exposed to drought stress commencing at the vegetative stage, our results add to this knowledge by showing the importance for seed yield of differential water extraction patterns when drought stress was also imposed at early podding. The more conservative water uptake at the early stage of reproductive growth resulted in more water being available during later reproductive growth for podding and seed growth. This is clear in Neelam that had more pods and seeds than the other genotypes in the WS treatment.

The different dynamics of water use in Neelam compared with the other genotypes during the reproductive phase resulted from lower canopy transpiration during the initial stage of the water draw-down and could be due to differences in leaf area and/or leaf stomatal conductance (Kholova et al., 2010; Kashiwagi et al., 2015; Pushpavalli et al., 2015). Stomatal conductance and leaf area were not measured in the present study, but the stomatal conductance was measured in DICC8156 and DICC8172 (two of the genotypes in this study) in an adjacent study in the glasshouse. In both these genotypes the stomatal conductance began to decrease when FTSW fell below 0.6, consistent with the threshold value for the decrease in transpiration and consistent with similar rates of transpiration (Pang et al., 2017). Further, the aboveground dry weight of Neelam at maturity did not differ significantly from six of the nine other genotypes in the WW treatment and from all others in the WS treatment, suggesting that the leaf area may not have been lower in Neelam during the growth period, and implying that the reduction in transpiration mainly arose from a reduction in stomatal conductance. This is consistent with the observations of Zaman-Allah et al. (2011b) that chickpea genotypes which used less water during the vegetative stage had lower canopy conductance, but contrasts with an earlier study that found no differences in leaf gas exchange among six chickpea genotypes that differed in yield when subjected to terminal drought in the field (Leport et al., 1999).

### Genotypic differences in the FTSW threshold for cessation of flowering and seed set

As far as we are aware, the present study identified for the first time significant genotypic differences in the threshold FTSW for the cessation of flowering and seed set in chickpea. The threshold FTSW for the cessation of seed set was higher (0.12–0.53 FTSW) than the cessation of flowering (0.08–0.28 FTSW), as found in grasspea (Kong et al., 2015) and also previously in two genotypes of chickpea (Pang et al., 2017). Thus, chickpea maintained flower production at much lower soil water contents and much longer into a drying cycle than seed set, even though the stomata had partially closed and photosynthesis had decreased (Pang et al., 2017). The FTSW at which flowering ceased varied from 0.28 in Neelam to 0.08 in DICC9073 and DICC8218. While the relationship between the FTSW at which flowering stopped and yield was not significantly different among the genotypes, it is interesting to note that the FTSW-value at which flower production ceased in Neelam, the highest yielding genotype in the WS treatment was higher than in the other genotypes, suggesting that cessation of flowering may be an effective way to reduce the carbon requirements for meristematic development and enable the resources to be allocated to existing pods and developing seeds.

The FTSW for the cessation of seed set varied between 0.12 and 0.53 in this study when drought stress was imposed at early podding. Pang et al. (2017) found that the FTSW at which the production of seeds ceased coincided with that at which leaf stomatal conductance and leaf transpiration rate also first decreased in chickpea. The FTSW for the cessation of seed set in the present study was in a similar range to FTSW threshold for the decline in leaf transpiration (0.25–0.43) when eight chickpea genotypes were grown outdoors and drought stress was imposed during the vegetative stage, while it was lower than FTSW threshold when the same eight genotypes were grown in a glasshouse (Zaman-Allah et al., 2011a). The FTSW for the cessation of seed set in our study was lower than FTSW for the decrease in leaf transpiration of 10 chickpea genotypes (0.44–0.83) when drought stress was also imposed during the reproductive stage (Pushpavalli et al., 2015). However, the relationship between the FTSW at which seed set ceased and yield was not significantly different among the genotypes, so its contribution to drought tolerance of chickpea genotypes is still to be established.

### Drought effects on the reproductive process

A positive linear correlation was evident between seed yield and seed number, seed yield and filled pod number in both the WW and WS treatments, consistent with other studies of chickpea; e.g., in Pushpavalli et al. (2015) with 10 genotypes. Flower production contributes to the seed yield in the WW treatment with a positive linear correlation between the two parameters, but no such relationship was found in the WS treatment, presumably because flower abortion and pod abscission affect the relationship between flower number and seed yield. Indeed, seed yield in the WS treatment had a negative linear correlation with the percentage of flower abortion and percentage of abscised pods, although no such relationship was found in the WW treatment, indicating that both flower and pod abortion were important factors reducing seed yield in the WS treatment. However, mean seed weight in the WS treatment did not differ significantly from that in the WW treatment in Neelam, DICC8218, and CICA0912 (*P* > 0.05), whereas it decreased in the other genotypes. The maintenance of seed size in these three genotypes during WS treatments could involve various mechanisms; in Neelam cessation of flowering at a higher FTSW could direct limited resources to fill existing pods, but these genotypes might also differ in the translocation of photosynthate from the leaves and stems to the early cohort of seeds that developed.

### Comparison of drought tolerance/sensitivity in the glasshouse with that in the field

The genotypes DICC8172 and WACPE2160 that were high yielding at the dryland field sites (Pang et al., 2017) also showed relatively high yields with terminal drought imposed in the glasshouse, while other genotypes DICC8218 and CICA0912 that were low yielding in the dryland field sites (Pang et al., 2017) also showed low yields in the WS treatment in the present glasshouse study. However, for some genotypes, seed yield in the glasshouse study ranked differently from that in the dryland field studies (Pang et al., 2017), indicating large genotype × environment interaction, as also found for other chickpea genotypes by Pushpavalli et al. (2015). As the environmental conditions at the field sites differed from those in the glasshouse, and even from field site to field site leading to variable results in some genotypes such as, Neelam, Genesis836, and DICC9100, the differences in seed yield ranking between the field and glasshouse were expected. In the field, although terminal drought is a common in spring under Mediterranean-type environment of southwestern Australia, there is often the occurrence of spring rainfall after podding as found in our field trials in both 2012 and in 2013 (<5 mm; Pang et al., 2017). In the glasshouse, no additional water was applied to the containers after the commencement of the terminal drought treatment. Our finding that chickpea maintained flower production at much lower soil water contents than seed set may enable opportunistic flower and pod set at field sites with typical Mediterranean-type environments which are characterized by erratic, unpredictable rainfall (Siddique et al., 1999). Thus, the small amount of spring rainfall may contribute to seed setting in the later-formed pods. Chickpea cultivar Neelam was bred and developed in Western Australia and has the potential to respond to spring rainfall in terms of new flowers and pods. Therefore, the differential capability of opportunistic reproduction in response to spring rainfall among chickpea genotypes may also partially explain the differences in yield ranking between the field and glasshouse studies (Pang et al., 2017). We suggest that glasshouse screening for drought tolerance among genotypes has limited applicability in the field, but provides an opportunity to identify traits associated with drought tolerance under more controlled and consistent conditions than the field, for possible use (together with field data) in selection of parents in breeding programs that aim to combine certain traits.

In summary, under WS during the reproductive stage (“terminal drought”) Neelam had the highest seed yield among 10 chickpea genotypes. Genotypes differed in the FTSW when flowering and seed set ceased in the WS treatment, but these threshold FTSW-values were not correlated with seed yield in the WS treatment, that is they were not correlated with drought tolerance. Among the 10 genotypes, when water was withheld during early podding, Neelam had the lowest daily transpiration rate during the first 6 days, but the highest rate in the 10–21 days after water was withheld. Thus, Neelam maintained the highest soil water content in the first 16 days, enabling pod production, seed growth and water use to continue longer into the drying cycle. We conclude that conservative water use is associated with higher yields in chickpea exposed to terminal drought commencing during early podding.

## Author contributions

JP, NT, YD, TC, and KS designed the experiments and contributed to the data interpretation. JP and YD undertook the experiment and collected the data. JP analyzed the data and drafted the manuscript. All authors contributed to the revision of the manuscript and approved the final manuscript.

### Conflict of interest statement

The authors declare that the research was conducted in the absence of any commercial or financial relationships that could be construed as a potential conflict of interest.
