# Supplementary material for: Pattern of Water Use and Seed Yield under Terminal Drought in Chickpea Genotypes
Source: Front Plant Sci. 2017 Aug 9;8:1375. doi: 10.3389/fpls.2017.01375 (PMC5552816; doi:10.3389/fpls.2017.01375)
Supplement: Supplementary file 1 [file DataSheet1.docx]

***Supplementary Table, Figure captions and Figures.***

**Pattern of water use and seed yield under terminal drought in chickpea genotypes**

Jiayin Pang*, Neil C. Turner, Yan-Lei Du, Timothy D. Colmer, Kadambot H.M. Siddique

*Corresponding author. Email: Jiayin.Pang@uwa.edu.au

**Supplementary Table S1.** The number of flowers, number of aborted flowers, number of abscised pods and number of empty pods in well-watered (WW) and water-stressed (WS) plants at physiological maturity. Data are means ± s.e.m. (*n*=4). For the parameters where genotype × water treatment interaction is significant, LSD values at *P* = 0.05 are given for the interaction; if the two-way interaction is not significant, the LSD_0.05_ values are given for the effects of genotype and/or water treatment. ns, no significant difference.

|  |  | Number of flowers (plant^–1^) | Number of aborted flowers (plant^–1^) | Number of abscised pods (plant^–1^) | Number of empty pods (plant^–1^) |
| --- | --- | --- | --- | --- | --- |
| Neelam | WW | 295 ± 23 | 81 ± 21 | 13 ± 2 | 48 ± 6 |
|  | WS | 179 ± 12 | 90 ± 16 | 19 ± 2 | 35 ± 4 |
| DICC8172 | WW | 239 ± 19 | 68 ± 7 | 19 ± 2 | 30 ± 10 |
|  | WS | 127 ± 3 | 69 ± 4 | 15 ± 3 | 13 ± 1 |
| WACPE2160 | WW | 188 ± 15 | 47 ± 9 | 11 ± 4 | 21 ± 6 |
|  | WS | 123 ± 24 | 64 ± 17 | 18 ± 3 | 13 ± 4 |
| Genesis836 | WW | 315 ± 15 | 94 ± 2 | 22 ± 5 | 65 ± 25 |
|  | WS | 251 ± 9 | 156 ± 9 | 38 ± 7 | 27 ± 5 |
| DICC9073 | WW | 253 ± 19 | 78 ± 8 | 29 ± 7 | 28 ± 7 |
|  | WS | 195 ± 23 | 120 ± 26 | 24 ± 2 | 23 ± 3 |
| DICC8156 | WW | 174 ± 18 | 33 ± 8 | 17 ± 4 | 21 ± 6 |
|  | WS | 64 ± 2 | 26 ± 2 | 9 ± 1 | 10 ± 1 |
| CICA1229 | WW | 269 ± 32 | 76 ± 8 | 15 ± 4 | 60 ± 10 |
|  | WS | 158 ± 22 | 84 ± 8 | 24 ± 5 | 26 ± 9 |
| DICC8218 | WW | 354 ± 43 | 117 ± 16 | 37 ± 8 | 57 ± 9 |
|  | WS | 243 ± 26 | 168 ± 15 | 28 ± 6 | 22 ± 3 |
| DICC9100 | WW | 287 ± 32 | 90 ± 17 | 17 ± 5 | 55 ± 8 |
|  | WS | 134 ± 8 | 88 ± 5 | 15 ± 2 | 13 ± 3 |
| CICA0912 | WW | 299 ± 22 | 87 ± 3 | 8 ± 2 | 52 ± 1 |
|  | WS | 141 ± 13 | 90 ± 14 | 21 ± 7 | 16 ± 1 |
| LSD_0.05_ | Genotype | 44 | 23 | 8 | 17 |
|  | Water | 20 | 10 | ns | 8 |
|  | Genotype × Water | ns | 32 | ns | 25 |

**Supplementary figure captions**

**Fig. S1.** Change in the fraction of transpirable soil water (FTSW) in the water-stressed treatment with time after the start of the water treatments (100 DAS) in six chickpea genotypes: WACPE2160, Genesis836, CICA1229, DICC8218, DICC9100 and CICA0912. The dashed line represents FTSW in the well-watered treatment, which was maintained at 1.0 by watering to 80% field capacity every two days. Data are means ± s.e.m. (*n*=4).

**Fig. S2.** Volumetric soil water content at six different soil depths: 0-0.1 m (A), 0.1-0.2 m (B), 0.2-0.3 m (C), 0.3-0.4 m (D), 0.4-0.5 m (E), and 0.5-0.6 m (F) with time after the start of the water treatments (100 DAS) in the water-stressed treatment in six chickpea genotypes: WACPE2160, Genesis836, CICA1229, DICC8218, DICC9100 and CICA0912. Data are means ± s.e.m. (*n*=4).

**Fig. S3.** Changes in the cumulative number of flowers, total pods, and seeds per plant with time (days) after sowing in well-watered (WW) and water-stressed (WS) treatments in six chickpea genotypes: WACPE2160 (A), Genesis836 (B), CICA1229 (C), DICC8218 (D), DICC9100 (E) and CICA0912 (F). Data are means ± s.e.m. (*n*=4). Arrows indicate the start of the water treatment.

**Fig. S4.** The split-line regression between the cumulative number of flowers and cumulative number of seeds per plant and the fraction of transpirable soil water in the water-stressed treatment in two chickpea genotypes with contrasting seed yield: Neelam (A) and CICA0912 (B) showing the break point values at which the slope of the fitted regression changed significantly. Data are means of four replicates. Note the break in the *y*-axis of CICA0912 (B).

Supplementary Fig. S1 Pang et al.

Supplementary Fig. S2 Pang et al.


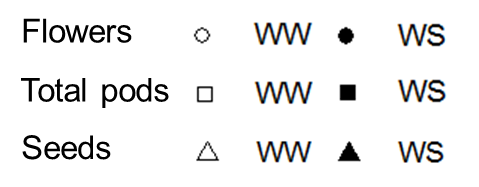


Supplementary Fig. S3 Pang et al.

Supplementary Fig. S4 Pang et al.
